# Temporal Association Between Exercise and Arrhythmia Episodes in Endurance Athletes With Paroxysmal Atrial Fibrillation

**DOI:** 10.1161/JAHA.126.050198

**Published:** 2026-09-29

**Authors:** Turid Apelland, Jon Magne Letnes, Guido Claessen, Kristel Janssens, Andreas Berg Sellevold, Rik Willems, Arnljot Tveit, Sophia Onarheim, Steve Enger, Sevda Ece Kizilkilic, Amy M. Mitchell, Hielko Miljoen, Adrian Elliott, Jan Pal Loennechen, Andre La Gerche, Marius Myrstad, the Norwegian Exercise and Atrial Fibrillation Consortium NEXAF

**Affiliations:** ^1^ Department of Medical Research Bærum Hospital Vestre Viken Trust Baerum Norway; ^2^ Faculty of Medicine, Institute of Clinical Medicine University of Oslo Oslo Norway; ^3^ Department of Cardiology and Cardiothoracic Surgery, St. Olav's Hospital Trondheim University Hospital Trondheim Norway; ^4^ Department of Circulation and Medical Imaging Norwegian University of Science and Technology (NTNU) Trondheim Norway; ^5^ Department of Cardiovascular Diseases KU Leuven Leuven Belgium; ^6^ Faculty of Medicine and Life Sciences, UHasselt Biomedical Research Institute Diepenbeek Belgium; ^7^ Department of Cardiology, Hartcentrum Hasselt Jessa Hospital Hasselt Hasselt Belgium; ^8^ Mary MacKillop Institute for Health Research ACU Melbourne Australia; ^9^ Heart, Exercise and Research Trial (HEART) Lab, St. Vincent's Institute of Medical Research Melbourne Australia; ^10^ Department of Cardiology University Hospitals Leuven Leuven Belgium; ^11^ Faculty of Medicine and Health Sciences Gent University Ghent Belgium; ^12^ Department of Medicine The University of Melbourne Fitzroy Australia; ^13^ Department of Cardiology University Hospital Antwerp Antwerp Belgium; ^14^ Department of Cardiovascular Sciences University of Antwerp Antwerp Belgium; ^15^ Centre for Heart Rhythm Disorders, University of Adelaide South Australian Health & Medical Research Institute and Royal Adelaide Hospital Adelaide Australia; ^16^ Department of Cardiology St. Vincent's Hospital Melbourne Fitzroy Australia; ^17^ HEART Lab Victor Chang Cardiovascular Research Institute Melbourne NSW Australia; ^18^ Department of Internal Medicine Bærum Hospital Vestre Viken Trust Baerum Norway

**Keywords:** atrial fibrillation, endurance athletes, exercise intensity, sports medicine, Atrial Fibrillation, Arrhythmias

## Abstract

**Background:**

Atrial fibrillation (AF) is a prevalent arrhythmia in endurance athletes, and many athletes report exercise as a trigger for AF episodes. We aimed to investigate the association between exercise sessions and arrhythmia episodes in endurance athletes with paroxysmal AF.

**Methods:**

In endurance athletes diagnosed with paroxysmal AF, we continuously monitored arrhythmia episodes using insertable cardiac monitors and report event rates per 24 hours and temporal relation of arrhythmias with exercise sessions. Sports watches and chest straps were used for exercise monitoring. Exercise with a heart rate of 76% to 84% and ≥ 85% of the individual maximum heart rate was classified as high and very high intensity, respectively.

**Results:**

Forty‐seven athletes (age 57±10, range 33–75 years, 3 women) were monitored with insertable cardiac monitors for a total of 55 994 hours (median 1008 [840–1344] equaling 42 days [35–56]) and performed a total of 1722 hours of exercise, of which 592 hours at high or very high intensity. Thirty‐one athletes (66%) had a total of 1445 (median 17 [3–33]) AF episodes during monitoring. Thirteen athletes (27%) had AF during exercise of whom 2 had AF during high‐intensity sessions. The event rate of AF was not higher during (0.34 per 24 hours [95% CI, 0.13–0.65]) or subsequently following an exercise session (0.37 [95% CI, 0.10–0.71]), compared with unrelated to exercise (>3 hours after, 0.61 [95% CI, 0.26–1.13]). There were no episodes of exercise‐related ventricular tachycardia.

**Conclusions:**

In this study on endurance athletes with paroxysmal AF, the rate of AF episodes was not increased during or subsequently following exercise sessions.

**Registration:**

URL: Study Details: NCT04991337. Effects of Detraining in Endurance Athletes With Atrial Fibrillation. ClinicalTrials.gov (NEXAF Detraining).

## Graphical abstract

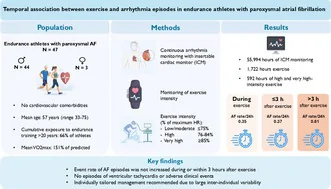

**Nonstandard Abbreviations and Acronyms**
CPET: cardiopulmonary exercise test
HRmax: maximum HR
ICM: insertable cardiac monitor

**Clinical Perspective**

**What Is New?**

- In this study on endurance athletes with paroxysmal atrial fibrillation (AF) continously monitored with insertable cardiac monitor, the rate of recurrent AF episodes was not increased during or within first 3 hours after exercise.
- During >1700 hours of exercise, including 592 hours of high‐ and very‐high‐intensity exercise, we observed only 1 high‐rate AF episode and no ventricular tachyarrhythmias or other clinical adverse events.

**What Are the Clinical Implications?**

- In endurance athletes with paroxysmal AF, arrhythmia episodes occurred both during and unrelated to exercise sessions, suggesting large between‐athlete variation and need for individually tailored management in terms of exercise recommendations; however, further studies with long‐term follow‐up are needed to assess the risks and benefits of continued endurance training in athletes with paroxysmal AF.

Regular physical activity is a cornerstone in primary and secondary prevention of cardiovascular morbidity and mortality. Compared with physical inactivity, light to moderate intensity physical activity reduces the risk of cardiac arrhythmias by ∼5% to 22%.^1^

Athletic individuals live longer than their less active peers in the general population,^2^, ^3^, ^4^ but paradoxically, prolonged vigorous exercise such as competitive endurance sport participation may promote cardiac arrhythmias.^5^ Although malignant arrhythmias and sudden cardiac death during sports are rare events and often attributed to underlying cardiac conditions,^6^, ^7^, ^8^ studies have revealed a high prevalence of paroxysmal atrial fibrillation (AF) in middle‐aged and older male endurance athletes.^9^, ^10^ Prolonged exposure to endurance exercise has been associated with an increased risk of incident AF.^11^ Although the underlying pathophysiological mechanisms remain understudied, AF may be attributed to exercise‐induced electrical and structural cardiac remodeling such as altered autonomic balance, atrial enlargement, and fibrosis.^12^, ^13^

One in 4 patients with AF in the general population report exercise as a trigger for AF episodes,^14^ and also among endurance athletes with AF exercise is a commonly reported trigger.^15^ At the same time, many athletes perceive themselves as healthy individuals and prefer to maintain high‐intensity sports activities even after having been diagnosed with AF.^15^, ^16^

Concerns about exercise‐induced AF episodes with high ventricular rate and the observation that vigorous endurance sports may possess proarrhythmic properties have contributed to a conservative approach regarding continuing high‐intensity exercise in patients with AF. Despite a lack of data on AF recurrence related to continuing high‐intensity exercise to inform decision‐making, athletes diagnosed with AF have typically been advised to decrease the intensity of their exercise.^17^, ^18^, ^19^

With the aim to investigate the temporal association between exercise sessions and arrhythmia episodes, we continuously monitored arrhythmia episodes with insertable cardiac monitors and studied the temporal association of arrhythmias with exercise sessions in endurance athletes diagnosed with paroxysmal AF.

## METHODS

### Study Design

The current study is an international multicenter prospective observational cohort study, based on data collected until randomization in the randomized controlled trial (RCT) NEXAF Detraining (Norwegian Exercise and Atrial Fibrillation Effects of Detraining in Endurance Athletes With Atrial Fibrillation, ClinicalTrials.gov Registry Identifier NCT04991337).^20^ The aim of the ongoing RCT is to study whether a 16‐week period with individually tailored training adaption could reduce AF burden in endurance athletes with paroxysmal AF and no cardiovascular comorbidities. We consecutively included the first 47 study participants enrolled in the period January 5, 2022 to November 27, 2024, at the 5 study centers: Bærum Hospital, Bærum, Norway; St.Olavs Hospital, Trondheim, Norway; Baker Heart and Diabetes Institute, Melbourne, Australia; Jessa Hospital Hasselt, Belgium; and the University Hospitals of Leuven, Belgium. The current substudy was prespecified in the RCT protocol. At inclusion, after signed informed consent, all athletes received an insertable cardiac monitor (ICM), a sports watch and a chest strap. Data included in this substudy were collected during a period of minimum 5 weeks from inclusion until randomization and thus before the intervention period of the RCT. Data may be shared upon reasonable request to the senior author after the NEXAF Detraining Trial is completed. The research reported in this paper adhered to the Strengthening the Reporting of Observational Studies in Epidemiology guidelines.

### Study Population

Endurance‐trained individuals reporting ≥5 hours/week of running/rowing or ≥8 hours/week of cycling, cross‐country skiing, or comparable sports, diagnosed with paroxysmal AF and reporting ≥2 episodes of AF of which 1 episode was during the past 6 months, were eligible for inclusion. Individuals who approached or were referred to 1 of the study centers were screened and included if they fulfilled all inclusion criteria and none of the exclusion criteria. Table 1 shows the eligibility criteria of the study. Individuals with a strong family history of coronary artery disease or sudden death, or unexplained symptoms during activity were excluded after preparticipation screening.^21^

**Table 1:** Study Eligibility Criteria for the Effects of Detraining in Endurance Athletes With Atrial Fibrillation Trial^20^

Inclusion criteria
Signed informed consent
2 Age ≥18 y
3 Diagnosed with paroxysmal atrial fibrillation (verified by ECG)
4 Athletes reporting ≥5 (running, rowing) or ≥8 (cycling, cross‐country skiing) weekly h, respectively, of endurance sport practice
5 At least 2 anamnestic (self‐reported) episodes of atrial fibrillation, of which 1 during the past 6 mo
6 Use a smartphone and agree to connect their sports watch with a web‐based platform for monitoring of exercise
Exclusion criteria
Permanent atrial fibrillation
2 Cardiac conditions (including valvular heart disease of moderate or greater severity, symptomatic ischemic heart disease)
3 Suspicion of undiagnosed ischemic heart disease
4 Left ventricular ejection fraction <45%
5 Arterial hypertension (>140/90 mm Hg)
6 Diabetes
7 Hyperthyroidism
8 Smoking during the past 5 y
9 Alcohol intake >20 alcohol units/wk
10 Use of illegal or performance enhancing drugs
11 Body mass index >30 kg/m^2^
12 Injuries preventing physical exercise
13 Pregnancy
14 Participation in conflicting intervention research studies
15 Planned atrial fibrillation ablation within the next 6 mo
16 The individual refuses to have an insertable cardiac monitor, blood samples taken, or be part of the detraining group
17 Sleep apnea

### Cardiac Arrhythmia Monitoring and Adverse Events

All study participants received a wireless ICM (Confirm Rx, Abbott, Sylmar, CA, USA) for continuous monitoring of cardiac arrhythmias and were monitored from implantation until randomization in the NEXAF Detraining Trial. The ICM was programmed to detect AF episodes ≥30 seconds and tachycardias, defined as ≥12 consecutive beats with a heart rate (HR) ≥180 beats per minute (bpm). In participants with a maximum HR (HRmax) >170 bpm, the ICM was programmed to detect tachycardia when HR exceeded the HRmax by ≥10 bpm. The ICM connected via Bluetooth and a smartphone application, and the data were uploaded to a secure server every 24 hours. Summarized reports of data from the ICM were regularly generated and contained episodic cardiac electrograms, date and time (hh:mm:ss) of the start and end of each episode, and duration and average HR of AF episode. Experienced cardiologists (T.A., G.C., A.L.G.) validated all arrhythmia episodes with available cardiac electrograms, except from AF episodes starting within 30 minutes following an already validated AF episode. AF episodes were classified as high‐rate AF if the HR was ≥180 bpm or the HR exceeded the individual HRmax by 10 bpm, in participants with a HRmax >170 bpm. The ICMs were programmed not to detect premature ventricular contractions or premature atrial contractions due to limited internal device memory.

We also studied arrhythmia episodes during (with a 12‐lead ECG) and subsequently following (with ambulatory 24‐hour ECG recording) a cardiopulmonary exercise test (CPET) to maximal effort.

We analyzed the temporal association between exercise sessions and recurrent AF episodes using date and time for the start and end of training sessions from sport watch recordings and date and time for the start and end of arrhythmia episodes from ICM recordings. Each AF episode was counted as a separate event. We classified episodes as AF occurring “during exercise” (onset during exercise), “subsequently following exercise” (onset within 3 hours following the end of an exercise session), and “unrelated to exercise” (onset >3 hours after exercise).

Any serious adverse clinical events during the study period were recorded. Serious adverse events were defined as death, any life‐threatening event, or any inpatient hospitalization.^22^

### Exercise Monitoring

We instructed the study participants to wear an HR chest strap (Garmin HRM‐PRO) and connected sports watch (Garmin Forerunner 745) during all exercise sessions throughout the study period. The HR monitor and watch connect using Bluetooth technology. Training sessions were uploaded automatically to a web‐based digital platform (Fitrockr, Digital Rebels, Berlin, Germany or TrainingPeaks, Louisville, CO, USA), where the exercise recordings were available to the researchers. Exercise session reports contained date and time (hh:mm:ss) of the start and end and the duration of each session.

HR was continuously recorded during exercise sessions. We defined the individual HRmax as the highest reliable HR measured with HR chest strap and a connected sports watch during a training session in the study period or measured in sinus rhythm during CPET. Training intensity was defined by the HR during training divided by the individual maximum HR ×100 and is reported as percentages of HRmax.

The recordings from each training session contained the number of seconds in predefined intensity zones. We classified exercise as low to moderate intensity (HR ≤75% of the individual HRmax), high intensity (HR 76%–84% for a minimum of 10 minutes), and very high intensity (≥85% of HRmax for a minimum of 10 minutes).^20^ All exercise sessions with high‐ and very‐high‐intensity exercise recordings were manually reviewed and cross‐checked with ICM recordings to ensure that the high HR during exercise represented exercise‐induced sinus tachycardia.

### Participant Characteristics

Body height and weight and physical fitness were measured during a study visit at the end of the ICM monitoring period of the current study (date of randomization in the NEXAF Detraining Trial). Participants completed questionnaires regarding their cumulative exercise years; weekly endurance hours during the past 6 months; primary endurance sport; frequency, duration, and triggers of AF episodes; current medication; and previous ablation procedures. Due to the strict eligibility criteria, age alone determined the athletes' CHA_2_DS_2_‐VA‐score. We performed transthoracic echocardiography in sinus rhythm at rest, following a prespecified protocol. Physical fitness was measured in sinus rhythm during CPET and is reported as maximal oxygen consumption (VO_2_max in ml/kg/min).^20^

### Statistical Analysis

We present continuous variables as mean±SD for normally distributed variables, median with interquartile range for nonnormally distributed variables, and categorical variables as frequencies and percentages (%). Event rates for recurrent AF episodes were calculated as the number of episodes per 24 hours of monitoring and are presented for each study participant, and for the total study cohort with 95% CIs based on bootstrapping using 500 iterations. We calculated the rate of AF episode for each time period of interest by dividing the number of AF episodes by the time under observation. Differences in the rates of AF between time periods were subsequently compared using a paired Wilcoxon signed‐rank test. To examine the temporal association between exercise and AF, postexercise time windows were categorized as ≤3 hours and >3 hours after exercise. The >3‐hour time period represented all AF episodes occurring in the time window starting 3 hours after exercise until the next exercise session started. The selection of these time periods was pragmatic and chosen as a balance between maximizing available AF event data and thereby reducing statistical uncertainty while maintaining clinically meaningful time windows for exploring potential underlying mechanisms. To assess the robustness of this categorization, supplementary analyses were performed using 1‐hour intervals following exercise. A generalized linear mixed‐effects model with a Poisson distribution and log link was fitted to estimate the association between time period and AF episode rates. Time period was included as a categorical fixed effect, participant ID as a random intercept, and the log of observation time was included as offset. A 2‐tailed *P* value <0.05 was considered statistically significant. All analyses were conducted using R version 4.4.1 (R Foundation for Statistical Computing, Vienna, Austria) or Ledidi Core version de7e4cc (Ledidi Core, Oslo, Norway).

### Ethical Considerations

The NEXAF Detraining Trial was approved by the relevant committees for medical research Committee in Norway (REK sør‐øst A/212748), Australia (HREC/76210/Alfred−2021 Project No. 470/21) and Belgium (Ethics Committee Research UZ/KU Leuven/ S65930), with the current analysis described as predefined substudy. All participants signed an informed consent to participate in the study.

## RESULTS

### Characteristics

A total of 47 athletes (3 women) with a mean age of 57±10 (range 33–75) years were included, of whom 36 (77%) were currently still engaged in competitive endurance sports on nonelite levels. The athletes reported an average of 6.8±2.3 weekly hours of endurance exercise and presented with high levels of cardiorespiratory fitness (mean VO_2_max 44±7 mL/kg/min), averaging 151±23% of predicted values, and 2 out of 3 reported a cumulative training load of ≥20 years. The most reported primary sports were cycling (38%), cross‐country skiing (26%), and running (26%). Ten athletes (21%) had a CHA_2_DS_2_‐VA score of 1 due to age >64 years, and 1 (2%) a CHA_2_DS_2_‐VA score of 2 due to age 75. Six athletes (13%) had recurrent AF after previous AF ablation. None of the participants used permanent rate or rhythm control medications during the study period. Table 2 shows the participants' characteristics, including selected echocardiographic parameters demonstrating well‐preserved cardiac function and cardiac remodeling typical for endurance athletes.^13^, ^23^ Left ventricular end‐diastolic volume index was only slightly increased, in men 76±13 mL/m^2^ and in women 60±5 mL/m^2^. Left atrial end‐systolic volume index was more elevated in men versus women (50±13 mL/m^2^ versus 37±6 mL/m^2^). Right atrial end‐systolic volume index was increased both in men (43±12 mL/m^2^) and women (39±9 mL/m^2^).

**Table 2:** Clinical and Echocardiographic Characteristics of Endurance Athletes With Paroxysmal Atrial Fibrillation Included in the Study (n=47)

Characteristics	No.=47*
Age, y	57±10
Body height, (cm)	182±7
Body mass index, kg/m^2^	24.1±2.3
Systolic blood pressure, mm Hg	128±12
Diastolic blood pressure, mm Hg	76±9
Resting heart rate, beats per min	52±8
Cumulative training load, y†	Participants, n (%)
	5 (11)
10–19	11 (23)
20–29	3 (6)
30–39	11 (23)
≥40	17 (36)
Echocardiographic variables	
LVEDV mL	151±33
LVEDV index, mL/m^2^	75±13
LV GLS, %	−20±2
LV GLS>−16%,‡ n (%)	0.0
LV ejection fraction, %	60±5
LAESV, mL (MOD)	102±31
LAESV indexed for BSA, mL/m^2^	50±12
MV, E/A‐ratio	1.6±0.4
MV E/e'	6±1
Tricuspid valve max velocity, m/s	2.3±0.4
Right atrial area, cm^2^	27±5
RAESV, mL (MOD)	87±26
RAESV indexed for BSA, mL/m^2^	43±11
Left atrial/left ventricular volume ratio§	0.7±0.2
LVEDV/VO_2_max indexǁ	43±7

BSA indicates body surface area; LAESV, left atrial end‐systolic volume, by machine mode; LV GLS, left ventricular global longitudinal strain, average; LVEDV, left ventricular end‐diastolic volume; MOD, The Observational cohort study; MV, mitral valve; and RAESV, right atrial end‐systolic volume, by machine mode.

* Data are presented as mean±SD or n (%).

^†^ Self‐reported number of years with regular endurance exercise.

^‡^ Number and percentage of athletes with LV GLS % above normal limit indicating reduced GLS.

^§^ Ratio based on LAESV and LVEDV.

^ǁ^ Ratio based on left ventricular end‐diastolic volume and the absolute VO_2_max‐value.

### Exercise Sessions

The athletes were monitored for a total of 55 994 hours, median 1008 hours (840–1344) equaling a median of 42 days (35–56). During the study period, the athletes performed a total of 1500 (mean per athlete 32±19) exercise sessions and a total of 1722 hours of exercise, of which 1130 (65%) hours were at low to moderate intensity, 354 hours (21%) at high intensity and 238 hours (14%) at very high intensity (Figure 1).

**Figure 1:**
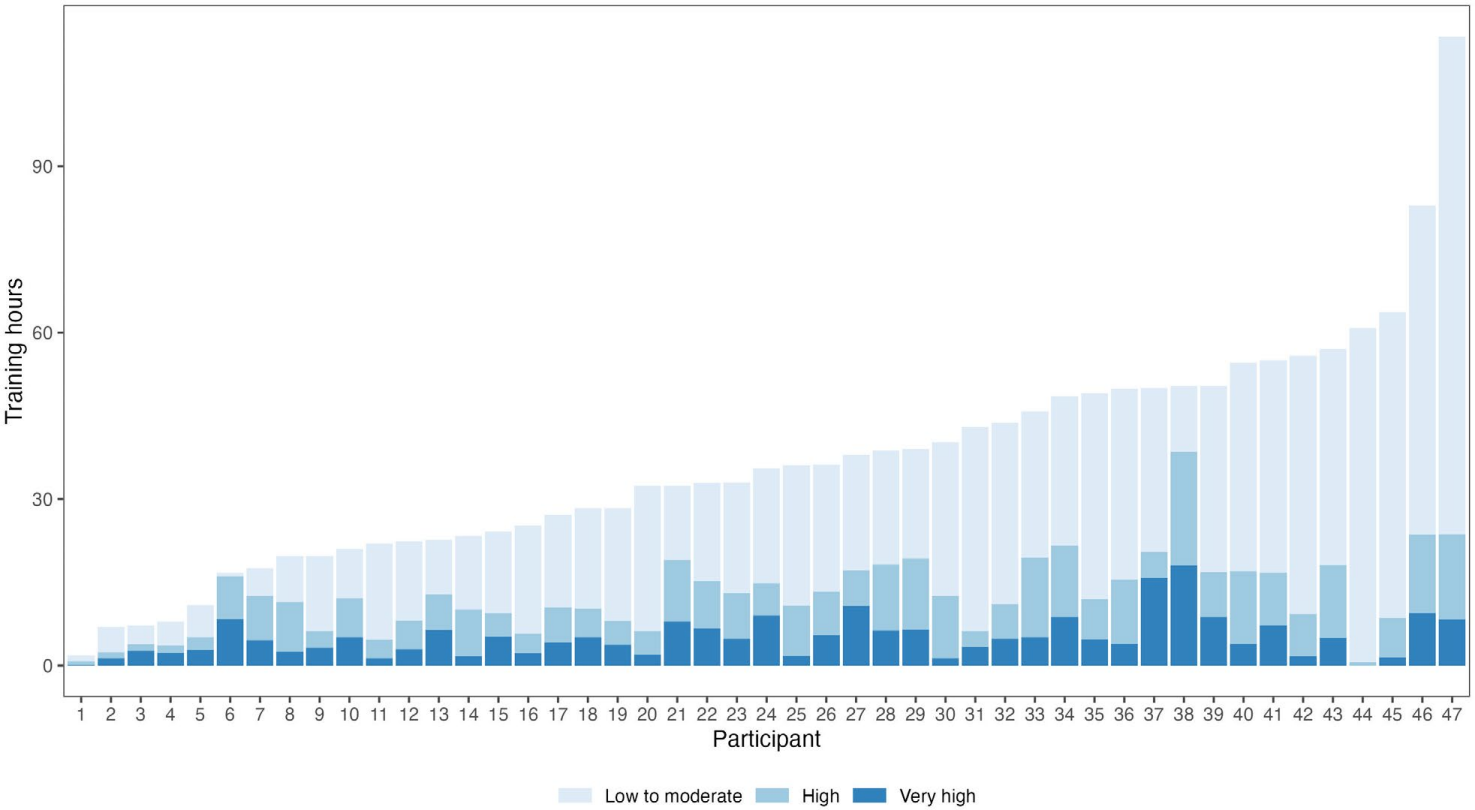
Hours of exercise during a median period of 42 days at low‐ to moderate‐ (HR ≤75% of HRmax), high‐ (HR 76–84% of HRmax) and very‐high‐ (HR ≥85% of HRmax) intensity among endurance athletes with paroxysmal atrial fibrillation (n=47) in the study. HR indicates heart rate.

### Recurrent Atrial Fibrillation Episodes

We observed a total of 1445 AF episodes (mean 31±3, median 3 [0–21] among 31 (66%) athletes in the study). Among athletes who experienced at least 1 AF episode during the study period, the mean number of episodes was 47±86 (median 17 [3–33]). There were 152 AF episodes (11%) with a duration >60 minutes and the median duration of AF episodes was 3 minutes (1.2–13.0). Fifteen episodes were classified as high‐rate AF. Only 1 short high‐rate AF episode occurred during exercise, which was a low‐ to moderate‐intensity exercise session.

In total, 13 individuals (28%) experienced recurrent AF during exercise, of whom 11 also had AF episodes unrelated to exercise. The median duration of AF episodes during exercise was 2.4 (1.2–8.2) minutes, mean 20±55 minutes. Two participants experienced AF only during exercise. Each of them had only 1 AF episode during the total study period in which they performed a total of 39 and 21 hours of exercise, respectively. In both cases, AF started during high‐intensity exercise; however, none of the episodes were high‐rate episodes.

Table 3 shows numbers and event rates for recurrent AF episodes during exercise, subsequently following within 3 hours after exercise, and unrelated to exercise (>3 hours after exercise). Eighteen athletes (38%) performing an average of 33±18 hours of exercise had AF episodes that were unrelated to exercise.

**Table 3:** Numbers (%), Event Rates, and Incidence Rate Ratio of Recurrent AF Episodes During Exercise, Subsequently Following Within 3 Hours After Exercise, and Unrelated to Exercise (>3 Hours After Exercise) During 55 944 hours of Continuous Monitoring With Insertable Cardiac Monitor in 47 Endurance Athletes Diagnosed With Paroxysmal Atrial Fibrillation

Monitoring period	AF episodes n (%)	Event rate (per 24 h) with 95% CI*	Incidence rate ratio with 95% CI
During a training session	35 (2.4)	0.35 (0.13–0‐65)	0.64 (0.46–0.9) *P*=0.0092
≤3 h after a training session	82 (5.7)	0.37 (0.10–0.71)	0.60 (0.48–0.75) *P*≤0.001
Unrelated to exercise (>3 h after exercise)	1328 (92)	0.61 (0,26–1.13)	1 (reference)

AF indicates atrial fibrillation.

* CI.

For the whole group the event rate of recurrent AF was not higher during or subsequently following exercise compared with >3 hours after exercise (Figure 2).

**Figure 2:**
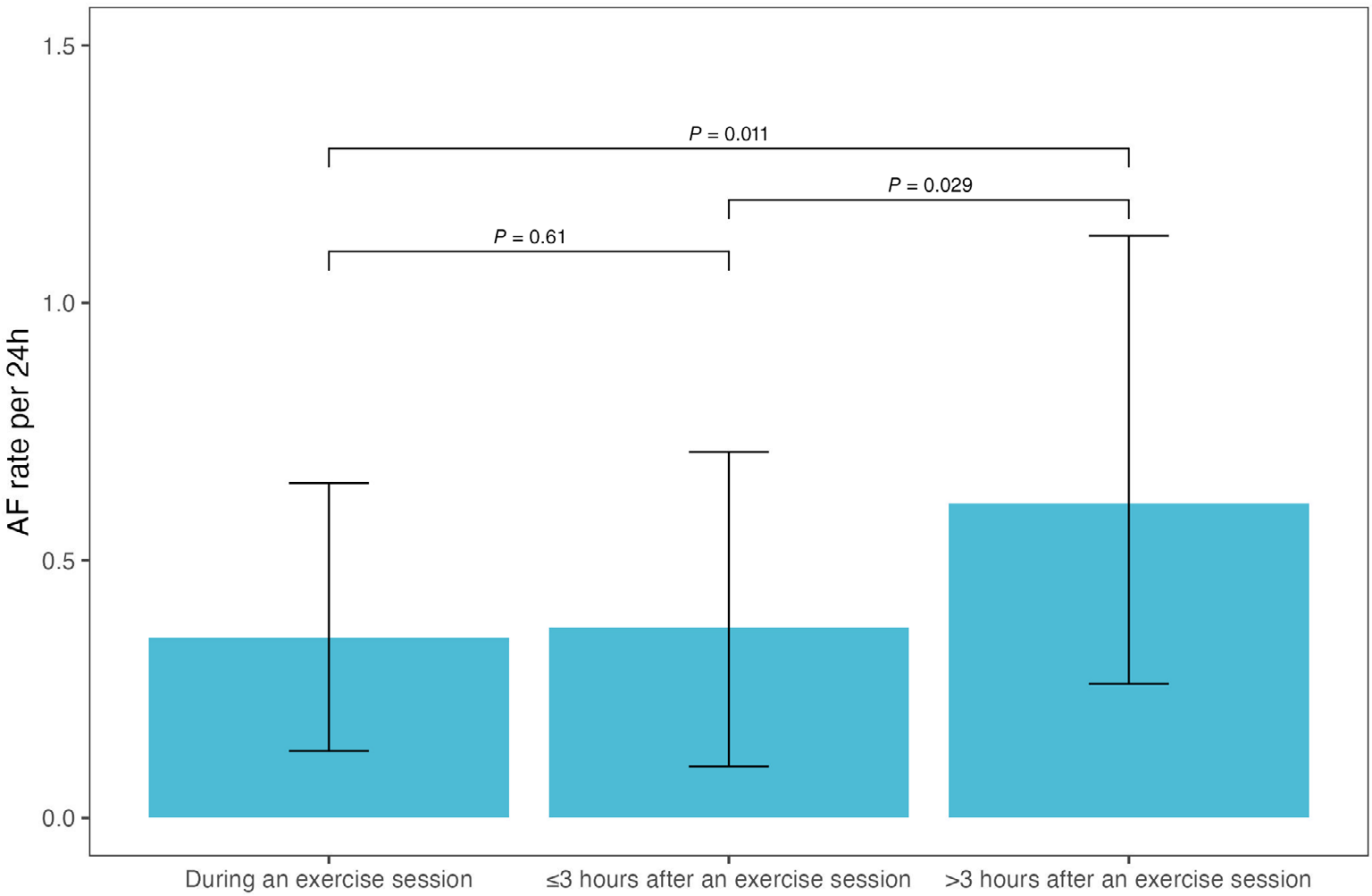
Event rates of recurrent atrial fibrillation episodes during exercise, within 3 hours after exercise, and unrelated to exercise (>3 hours after exercise), with 95% CIs and *P* values for comparison between categories among endurance athletes with paroxysmal atrial fibrillation (n=47). The athletes experienced a total of 1445 episodes of atrial fibrillation during a total of 55 944 hours. During this period the athletes performed a total of 1500 training sessions. AF indicates atrial fibrillation.

The number of AF episodes and event rates among the 31 athletes who had at least 1 episode of recurrent AF during the study period are shown in Table S1. Supplementary analyses using 1‐hour intervals following exercise demonstrated that the estimated AF rates were broadly consistent across consecutive hourly periods, suggesting that the choice of time window length did not materially influence the observed associations (Table S2).

AF recurrence event rates during, subsequently following, or unrelated to exercise for each of the 31 participants with at least 1 AF episode are shown in Figure 3. There was no association between degree of individual AF burden and the individual timing of AF recurrence.

**Figure 3:**
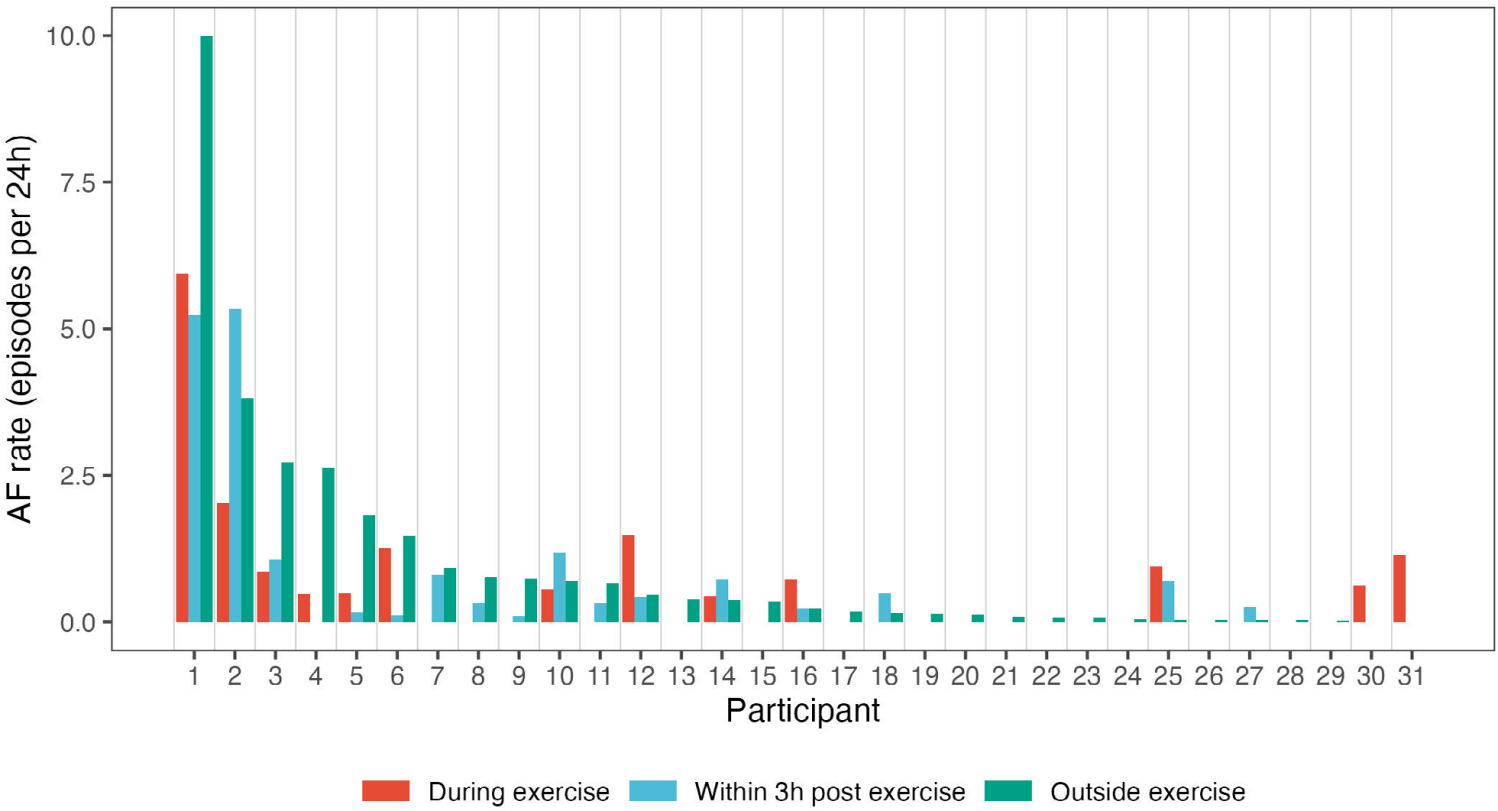
Participant level event rates of AF episodes per 24 hours during exercise, within 3 hours after exercise, and outside exercise in 31 athletes with at least 1 AF episode during the study period. AF indicates atrial fibrillation.

Table 4 shows self‐reported triggers for recurrent AF episodes assessed by questionnaires. Twenty‐eight athletes (60%) reported exercise as a typical trigger for their AF episodes, and 36 athletes (77%) reported that AF episodes were typically triggered by exercise or occurred directly after exercise sessions. Of the 28 athletes reporting exercise as a typical trigger, 11 (39%) also reported to have AF recurrence at night.

**Table 4:** Self‐Reported Triggers for Atrial Fibrillation Recurrence in Endurance Athletes Diagnosed With Paroxysmal Atrial Fibrillation (n=47). Some Athletes Reported Several Triggers

Triggers for atrial fibrillation	No. (%)
During exercise sessions	28 (60)
Directly after exercise sessions	16 (34)
During the night	20 (43)
During mental stress	13 (28)
During or after alcohol intake	14 (30)
After large meals	3 (6)
During infections/fever	5 (11)
Without any relation to the situations mentioned	10 (21)

### Ventricular Tachycardia and Adverse Clinical Events

We did not observe any exercise‐related ventricular tachycardia (VT) or serious adverse clinical events during the monitoring period. Neither did we observe any VT episodes or adverse clinical events during the 47 CPETs to maximum effort that were performed in the study.

Table S3 shows arrhythmia episodes recorded during 24‐hour ECG monitoring following CPET (in total 1128 hours of monitoring). One athlete had 2 episodes at rest of nonsustained VT of 4 and 6 complexes, respectively, which were not detected by the ICM due to the low rate of mean 96 and 138 bpm, respectively, and short duration. This athlete had no clinical events during the study period, presented with a normal 12‐lead ECG, unremarkable echocardiography findings, only 2% premature ventricular contractions during 24‐hour ECG monitoring, and an extraordinarily high cardiorespiratory fitness of 173% of predicted VO_2_max.

The 24‐hour ECG monitoring detected a total of 4 AF episodes in 4 athletes. One athlete presented with >500 premature ventricular contractions per 24 hours.

## DISCUSSION

In this study using continuous monitoring with ICM, endurance athletes diagnosed with paroxysmal AF performed >1700 hours of exercise, including almost 600 hours of high‐ or very‐high‐intensity exercise. The rate of recurrent AF episodes was not higher during or first 3 hours after exercise, compared with the monitoring periods without relation to exercise. Exercise did not trigger AF episodes with fast ventricular response, and we did not observe any severe adverse clinical events or episodes of VT during exercise.

Concerns about potential deleterious effects, including recurrent AF and AF episodes with high ventricular rate during high intensity exercise, have led to a cautious approach to exercise recommendations among many physicians. In contrast, both clinical experience and findings from previous studies suggest that many athletes perceive themselves as healthy individuals and wish to continue sports participation and high‐intensity exercise even after an AF diagnosis.^15^ Consequently, in otherwise healthy and athletic individuals, typically presenting with relatively short and rare arrhythmia episodes, conflicting interests such as the desire to uphold high‐intensity exercise and exercise restrictions due to safety concerns may cause tensions in the doctor–patient relationship.^24^ Notably, despite 60% of the athletes reporting exercise as a typical trigger for AF recurrence, the continuous monitoring revealed that only 28% had AF during exercise. The mismatch between self‐reported exercise‐triggered AF and the lower rate of AF episodes that actually occurred during exercise may be related to the relatively short monitoring period. However, the discrepancy may also be related to recall bias with AF episodes occurring during exercise being perceived more severe with higher consequence and impact on quality of life. Furthermore, some athletes may have AF episodes being too short to be detected by the ICM (<30 seconds). Alternatively, the palpitations do not represent AF but for instance frequent premature atrial contractions or short paroxysmal supraventricular tachycardias that the ICM is not programmed to store.

Among the 60% of athletes reporting exercise as a typical AF trigger, 39% also reported prevalent AF recurrence at night. We found that among the 28% of participants with ICM‐detected AF during exercise, a majority also had AF outside exercise. However, there were great interindividual variations regarding AF recurrence patterns as shown in Figure 3. These findings demonstrate the complexity of AF triggers in athletes. Furthermore, our observations underline the limitation of self‐reporting of AF episodes and suggest that monitoring of exercise and arrhythmia episodes may be useful as part of the management of endurance athletes with AF.

A striking observation in this study is the large variations between athletes. Arrhythmia episodes occurred both during and unrelated to exercise sessions, highlighting the need for individually tailored management in terms of exercise recommendations in athletes diagnosed with AF.

Study participants were middle‐aged or older athletes of ∼45 to 65 years of age. In this age group, previous studies on athletes have reported a strikingly high prevalence of AF of 5% to 21%.^11^, ^25^, ^26^, ^27^ The management of this particular subgroup of patients with AF is challenging and largely based on clinical experience and expert opinion. Despite recent advances, treatment options remain unsatisfactory for many athletes, with antiarrhythmic drugs not being well tolerated in athletic individuals. Pulmonary vein ablation is an effective first‐line treatment in highly symptomatic individuals.^28^ However, AF recurrence after ablation remains an issue in a relatively large proportion of the patients.^29^, ^30^ Exercise intensity modification may be an option in symptomatic athletes, especially when AF episodes are often triggered by high‐intensity exercise. The ongoing randomized controlled trial NEXAF Detraining will provide more data on the effect of individually tailored exercise adaption, including reduced exercise intensity in athletes diagnosed with AF.^20^

Although the current study adds novel insights into the temporal association between exercise and recurrent AF episodes, the underlying mechanisms are not fully understood.^31^ In some individuals AF seems to be triggered by exercise, potentially through sympathetic activation, whereas other athletes in our study had AF unrelated to exercise. A relatively high proportion reported that AF often occurred during night, indicating that parasympathetic activation may have played a role in this subset of the studied athletes. Interestingly, almost 40% of the athletes who reported exercise as a trigger for AF also reported having AF episodes at night, underlining the complexity of both the underlying mechanisms and of AF management.

Moreover, this is also an age group in which the risk of sports‐related sudden cardiac death is considerably higher compared with younger athletes.^32^ Further prospective studies with prolonged follow‐up and clinical end point evaluation are warranted to establish whether high‐intensity exercise and participation in endurance sports are safe in athletes diagnosed with AF.

Only 1 athlete had a high‐rate AF episode during exercise, which was a low‐moderate intensity session. No high‐rate AF episodes occurred during high‐ or very‐high‐intensity sessions. Two athletes experienced 1 AF episode each during the total study period. Both episodes occurred during a high‐intensity session but were not high‐rate AF, and none of the athletes had any further AF episodes despite a total of 39 and 21 hours of exercise, respectively.

In 1 of the athletes in the study, we observed 2 short episodes of nonsustained VT at rest. In a previous study, nonsustained VT was observed in 3% of otherwise healthy athletes (mean age 45 years) monitored with 24‐hour ECG. In that study the occurrence of nonsustained VT was comparable to that of a nonathletic healthy control group.^33^ Short nonsustained VTs are occasionally seen in athletes, with unknown clinical relevance in the absence of symptoms or pathological findings during clinical examination.^23^

Lifestyle intervention is a pillar of AF management. Guidelines advocate that regular physical activity of moderate to high intensity should be prescribed to manage risk factors and comorbidities and may prevent both incident and recurrent AF.^34^, ^35^ RCTs have demonstrated that short‐term exercise interventions with moderate to high exercise intensity may improve cardiorespiratory fitness and AF‐related quality of life and reduce AF recurrence and symptoms in patients with AF with previously low physical activity levels.^36^, ^37^, ^38^ Data from randomized studies may aid the development of more specific recommendations for subgroups of patients with AF.

### Strengths and Limitations

The main strength of the current study is a thorough arrhythmia assessment using continuous monitoring with ICMs. Moreover, the participating athletes performed a total of >1700 hours of exercise during the monitoring period, including almost 600 hours with high‐ or very‐high‐intensity exercise. The intensity of exercise was objectively measured through HR data from chest straps and sports watches, providing high‐quality data for intensity estimation. Furthermore, the novelty of combining data from ICM and sports watches allowed new insight into the temporal association of exercise and recurrent AF episodes. The study cohort of athletes was well defined and consisted of individuals with AF following prolonged exposure to endurance exercise and no cardiovascular comorbidities, suggesting the study has captured the phenotype of exercise‐related AF.

The main limitations are the relatively small study size and the relatively short period of monitoring, which increase the risk of a Type II error. A more prolonged monitoring period may have revealed more arrhythmia episodes and provided the study with more power to detect different rates of arrhythmia episodes between the categories of exercise and to further decipher the temporal association of recurrent AF episodes with exercise of different intensities. On the other hand, the targeted cardiac examination, with both cardiac imaging, extended continuous monitoring, CPET to maximal effort, and ambulant ECG monitoring subsequently following CPET, represents a sufficient and highly specific assessment protocol for arrhythmia evaluation in athletes,^19^ with a presumably high sensitivity to detect clinically relevant arrhythmia episodes. The inability of the ICM to detect tachycardia shorter than 12 complexes is another limitation, and shorter nonsustained VT may have remained undetected. However, short nonsustained VT episodes are occasionally seen in athletes, with unknown clinical relevance in the absence of symptoms or pathological findings during clinical examinations.^39^

Importantly, because individuals with cardiovascular comorbidities were excluded from the study, the results should not be projected to other subgroups of patients with AF or to athletes with AF and concomitant cardiovascular conditions. All the athletes participating in the study underwent cardiovascular risk assessment before entering the study, limiting the generalizability of the results to unselected cohorts of athletes. Due to the eligibility criteria, both asymptomatic individuals and highly symptomatic athletes awaiting ablation therapy may be underrepresented in the study. Moreover, only 3 women were included. Cardiac arrhythmias are 5‐ to 10‐fold less common in female compared with male athletes.^1^, ^40^ Thus, reports from even small cohorts of women with exercise‐related cardiac arrhythmias remain important awaiting larger studies that could properly address gender differences in the risk related to sports participation. Due to the mentioned limitations of the study, we hope the ongoing RCT NEXAF Detraining Trial will provide new answers.

## CONCLUSIONS

In this study, endurance athletes with paroxysmal AF did not experience an increase in the rate of AF episodes during or within 3 hours following exercise sessions, and the vast majority of AF episodes were unrelated to exercise. Large between‐athlete variations underscore the need for individually tailored management in athletes with AF. During >1700 hours of exercise and almost 600 hours with high‐ or very high‐intensity exercise, we observed no serious adverse clinical events or VT among the athletes.

## Sources of funding

The study was funded by the Norwegian Health Association and Vestre Viken Hospital Trust, Norway.

## Disclosures

None.

## Supporting information

STROBE Statement

Tables S1–S3
