# Supplementary material for: Temporal Association Between Exercise and Arrhythmia Episodes in Endurance Athletes With Paroxysmal Atrial Fibrillation
Source: J Am Heart Assoc. 2026 Sep 29;15(19):e050198. doi: 10.1161/JAHA.126.050198 (PMC13744992; doi:10.1161/JAHA.126.050198)
Supplement: Supplementary file 1 [file JAH3-15-e050198-s001.pdf]

Supplemental Material

Table S1. Numbers (%) and event rates of recurrent atrial fibrillation (AF) episodes during exercise, subsequently following within three hours after exercise, and unrelated to exercise (>3 hours after exercise) during continuous monitoring with insertable cardiac monitor in 31 out of 47 endurance athletes diagnosed with paroxysmal AF experiencing at least one episode of AF during the study period.

**Monitoring period**	**Recurrent atrial fibrillation episodes n (%)**	**Event rates for recurrent atrial fibrillation episodes (per 24 h) with 95% CI**
During a training session	35 (2.4)	0.54 (0.24-1.03)
≤3 hours after a training session	82 (5.7)	0.56 (0.18-1.03)
Unrelated to exercise (>3 h after exercise)	1328 (92)	0.94 (0.40-1.73)

Table S2. Event rates of recurrent atrial fibrillation (AF) episodes using individual 1-hour intervals following exercise up to the sixth hour post exercise among 31 participants with any AF episode during the study period.

Hour post exercise	AF rate per 24 hours (95%CI*)
First	0.46 (0.12-0.93)
Second	0.25 (0.04-0.52)
Third	0.39 (0.12-0.74)
Fourth	0.32 (0.10-0.55)
Fifth	0.33 (017-0.53)
Sixth	0.54 (0.21-0.99)

*Confidence interval

Table S3. Cardiac arrhythmias detected during 24-hour ECG monitoring subsequently following cardiopulmonary exercise testing to maximum effort in endurance athletes with paroxysmal atrial fibrillation (n=47).

**Cardiac arrhythmia**	**Mean±SD n=47**	**Median (IQR) n=47**
Isolated premature atrial contractions, n*	436±719	99 (36-443)
Premature atrial contractions, %†	0.7±0.9	0.4 (0.06-1,5)
Paroxysmal supraventricular tachycardia, n	3±4.4	1 (1-4)
Premature ventricular contractions, n	138±345	28 (7-141)

Premature ventricular contractions, %	0.2±0.5	0.05 (0.01-0.2)
Paroxysmal atrial fibrillation, n	4±4.6	2.5 (1.3-9)
Non-sustained ventricular tachycardia, n	2±0[‡]	N/A[§]
Sustained ventricular tachycardia, n	0	0
**Number of participants with arrhythmias**	**n (%)**	
Atrial fibrillation episodes	4 (9)	
Atrial flutter episodes	1 (2)	
>100 Premature ventricular contractions	12 (26)	
>500 Premature ventricular contractions	1 (2)	
Non-sustained ventricular tachycardia	1 (2)	

Data are presented as mean (SD), median (Interquartile range) or number (%). *Number per 24-hour, †Percent of total beats, ‡Both episodes in same athlete at rest, lasting <2 seconds, §Not applicable.

List of Collaborators, NEXAF consortium

This work has been done within the framework of the Norwegian Exercise and Atrial Fibrillation Initiative (NEXAF) project (https://nexaf.no), which is a collaborative research initiative aiming to increase knowledge about physical activity and exercise in relation to atrial fibrillation.

The Norwegian Exercise and Atrial Fibrillation consortium

Bente Morseth[1,2], Marius Myrstad[3,4], Bjarne Martens Nes[5,6], Jan Pål Loennechen[5,6], Maja-Lisa Løchen[7], Arnljot Tveit[4,8], Turid Apelland[4,8], Kristine Folkenborg[4], Kristoffer Robin Johansen[1,2], Kristin Espolin Johnson[5], Jon Magne Letnes[5,6], Vegard Malmo[5,6], Andreas Berg Sellevold[5,6], Ingrid Haagenrud Langmo[5], Eivind Sørensen[3,4], Rune Byrkjeland[3,4], Steve Enger[4], Fedelix Pethogo Brown[9], Sophia Onarheim[4], Vigdis Bache-Semb[4], Lena Marie Kristiansen[9].

Affiliations

[1]School of Sport Sciences, Faculty of Health Sciences, UiT The Arctic University of Norway, Tromsø, Norway
[2] Centre for Research and Education, University Hospital of North Norway, Tromsø, Norway
[3] Department of Internal Medicine, Bærum Hospital, Vestre Viken Hospital Trust, N-1346 Gjettum, Norway
[4] Department of Medical Research, Bærum Hospital, Vestre Viken Hospital Trust, N-1346 Gjettum, Norway
[5]Department of Circulation and Medical Imaging, Norwegian University of Science and Technology, NTNU, Trondheim, Norway
[6]Department of Cardiology and Cardiothoracic Surgery, St. Olav's Hospital, Trondheim University Hospital, Trondheim, Norway.
[7] Department of Community Medicine, Faculty of Health Sciences, UiT The Arctic University of Norway, Tromsø, Norway
[8] Faculty of Medicine, Institute for Clinical Medicine, University of Oslo, Oslo, Norway
[9]Department of Cardiology, University Hospital of North Norway, Tromsø, Norway
