# Supplementary material for: Temporal Association Between Exercise and Arrhythmia Episodes in Endurance Athletes With Paroxysmal Atrial Fibrillation
Source: J Am Heart Assoc. 2026 Sep 29;15(19):e050198. doi: 10.1161/JAHA.126.050198 (PMC13744992; doi:10.1161/JAHA.126.050198)
Supplement: Supplementary file 2 [file JAH3-15-e050198-s002.pdf]

STROBE Statement—checklist of items that should be included in reports of observational studies

	Item No.	Recommendation	Page No.	Relevant text from manuscript
**Title and abstract**	1	(*a*) Indicate the study's design with a commonly used term in the title or the abstract	2	Indicated in the abstract: In endurance athletes diagnosed with paroxysmal AF, we continuously monitored arrhythmia episodes using insertable cardiac monitors (ICM)
		(*b*) Provide in the abstract an informative and balanced summary of what was done and what was found	2	Provided in the conclusion of the abstract.
**Introduction**				
Background/rationale	2	Explain the scientific background and rationale for the investigation being reported	4	Prolonged vigorous exercise such as competitive endurance sport participation may promote cardiac arrhythmias. Although the underlying pathophysiological mechanisms in this group remain understudied, prolonged exposure to endurance exercise has been associated with an increased risk of incident AF, and AF may be attributed to exercise-induced electrical and structural cardiac remodeling such as altered autonomic balance and atrial enlargement and fibrosis. The safety of high-

intensity exercise in individuals with cardiovascular diseases remains an important knowledge gap. One in four patients with AF in the general population report exercise as a trigger for AF episodes. Concerns about exercise-induced AF episodes with high ventricular rate and the observation that vigorous endurance sports may possess proarrhythmic properties, have contributed to a conservative approach regarding continuing high-intensity exercise in AF patients. There is a lack of data on safety and AF recurrence related to continuing high-intensity exercise to inform decision-making, but athletes diagnosed with AF have typically been advised to decrease the intensity of their exercise. At the same time, many athletes perceive themselves as healthy individuals and prefer to maintain high-intensity sports activities even after having been diagnosed with AF.

Objectives	3	State specific objectives, including any prespecified hypotheses	4	With the aim to investigate the safety and potential deleterious effects of endurance exercise, we prospectively studied the occurrence of adverse clinical events, ventricular tachycardia (VT), high-rate AF episodes, and the temporal association of exercise with recurrent AF episodes in endurance athletes diagnosed with paroxysmal AF. There were no prespecified hypotheses.
**Methods**				
Study design	4	Present key elements of study design early in the paper	5	Study design is described early.
Setting	5	Describe the setting, locations, and relevant dates, including periods of recruitment, exposure, follow-up, and data collection	5	The current study is an international multicenter prospective observational cohort study, based on data collected until randomization in the randomized controlled trial Effects of Detraining in Endurance Athletes with Atrial Fibrillation (The NEXAF Detraining Trial, ClinicalTrials.gov Registry Identifier NCT04991337). We consecutively included the 47 first study participants enrolled in the period 5 January 2022 to 27 November 2024, at the five

				study centers Bærum Hospital, Bærum, Norway, St.Olavs Hospital, Trondheim, Norway, Baker Heart and Diabetes Institute, Melbourne, Australia, Jessa Hospital Hasselt, Belgium, and the University Hospitals of Leuven, Belgium.
Participants	6	(*a*) *Cohort study*—Give the eligibility criteria, and the sources and methods of selection of participants. Describe methods of follow-up	5	Endurance-trained individuals reporting ≥5 hours/week of running/rowing or ≥8 hours/week of cycling, cross-country skiing, or comparable sports, diagnosed with paroxysmal AF and reporting ≥2 episodes of AF of which one episode during the past six months, were eligible for inclusion. Individuals who approached or were referred to one of the study centers were screened and included if they fulfilled all inclusion criteria and none of the exclusion criteria. Table 1 shows the eligibility criteria of the study. Individuals with a strong family history of coronary artery disease or sudden death, or unexplained symptoms during

				activity were excluded after preparticipation screening.
Variables	7	Clearly define all outcomes, exposures, predictors, potential confounders, and effect modifiers. Give diagnostic criteria, if applicable	6	Please see below. There were no interventions in the observational cohort study. Variables of interest are described in detail below.
Data sources/ measurement	8*	For each variable of interest, give sources of data and details of methods of assessment (measurement). Describe comparability of assessment methods if there is more than one group	7	**Cardiac arrhythmia monitoring and adverse events:** All study participants received a wireless insertable cardiac monitor (ICM, Confirm RxTM, Abbott, Sylmar, CA, United States) for continuous monitoring of cardiac arrhythmias and were monitored from implantation until randomization in the NEXAF Detraining Trial. The ICM was programmed to detect AF episodes ≥30 s and tachycardias, defined as ≥12 consecutive beats with a HR ≥180 beats per minute (bpm). In participants with a maximum HR (HRmax) >170 bpm, the ICM was programmed to detect tachycardia when HR exceeded

the HRmax by ≥10 bpm. The ICM connected via Bluetooth and a smartphone application, and the data was uploaded to a secure server every 24 hours. Summarized reports of data from the ICM were regularly generated and contained episodic cardiac electrograms, date and time (hh:mm:ss) of the start and end of each episode, and duration and average HR of AF episode. Experienced cardiologists (TA, GC, ALG) validated all arrhythmia episodes with available EGM, except from AF episodes starting within 30 min following an already validated AF episode. AF episodes were classified as high-rate AF if the HR was ≥180 bpm or the HR exceeded the individual HRmax by 10 bpm, in participants with a HRmax >170 bpm. The ICMs were programmed not to detect premature ventricular contractions or premature atrial contractions due to limited internal device memory.

We also studied arrhythmia episodes during (with a 12-lead

electrocardiogram, ECG) and subsequently following (with ambulatory 24-hour ECG recording) a cardiopulmonary exercise test (CPET) to maximal effort.

Exercise monitoring

We instructed the study participants to wear a HR chest-strap (Garmin HRM-PRO) and connected sports watch sports watch (Garmin Forerunner 745) during all exercise sessions throughout the study period. The HR monitor and watch connect using Bluetooth technology. Training sessions were uploaded automatically to a web-based digital platform (Fitrockr, Digital Rebels, Berlin, Germany or TrainingPeaks, Louisville, Colorado, USA), where the exercise recordings were available to the researchers. Exercise session reports contained date and time (hh:mm:ss) of the start and end, and duration of each session. We defined the individual HRmax as the highest reliable HR measured with HR chest-strap and a connected sports

watch during a training session in the study period or measured in sinus rhythm during CPET. Training intensity was defined by the HR during training divided by the individual maximum HR x100 and is reported as percentages of HRmax.

The recordings from each training session contained the number of seconds in predefined intensity zones. We classified exercise as low- to moderate-intensity (HR ≤75% of the individual HRmax), high-intensity (HR 76-84% for a minimum of 10 minutes) and very high-intensity (≥85% of HRmax for a minimum of 10 minutes) exercise[23]. All exercise sessions with high- and very high-intensity exercise recordings were manually reviewed and cross-checked with ICM recordings to ensure that the high HR during exercise represented exercise-induced sinus tachycardia.

Participant characteristics

Body height and weight and physical fitness were measured

				during a study visit at the end of the ICM monitoring period of the current study (date of randomization in the NEXAF Detraining Trial). Participants completed questionnaires regarding their cumulative exercise years, weekly endurance hours during the past six months, primary endurance sport, frequency, duration and triggers of AF episodes as well as current medication and previous ablation procedures. We performed transthoracic echocardiography in sinus rhythm at rest, following a pre-specified protocol. Physical fitness was measured in sinus rhythm during CPET and is reported as maximal oxygen consumption (VO2max in ml/kg/min).
Bias	9	Describe any efforts to address potential sources of bias	15	Despite efforts to include more women, few female athletes were included. This is described at page 15.
Study size	10	Explain how the study size was arrived at	5	We included all 47 participants who were enrolled in NEXAF Detraining and had attended the Baseline examinations at a specific time point.

Continued on next page

Quantitative variables	11	Explain how quantitative variables were handled in the analyses. If applicable, describe which groupings were chosen and why	7	As described above ICM-detected arrhythmias and 24h ECGs were analyzed and validated by experienced cardiologists. The athletes were not grouped in the published analyses, however in a supplementary table we show details from the athletes who had at least one AF episode during the monitoring period.
Statistical methods	12	(*a*) Describe all statistical methods, including those used to control for confounding	7	We present continuous variables as mean ± standard deviation (SD) for normally distributed variables, median [interquartile range (IQR)] for non-normally distributed variables, and categorical variables as frequencies and percentages (%). Event rates for recurrent AF episodes were calculated as the number of episodes per 24 hours of monitoring, with 95% confidence intervals based on bootstrapping using 500 iterations. The Wilcoxon signed-rank test was used to compare differences in AF rate during a training session compared ≤3 hours and >3 hours after a training session.<br>A two-tailed P value <0.05 was considered statistically significant. All analyses were conducted using R version 4.4.1 (R Foundation for

				Statistical Computing, Vienna, Austria) or Ledidi Core version de7e4cc (Ledidi Core, Oslo, Norway).
		(*b*) Describe any methods used to examine subgroups and interactions		
		(*c*) Explain how missing data were addressed		We did not have any missing data.
		(*d*) *Cohort study*—If applicable, explain how loss to follow-up was addressed		We did not have any loss to follow-up.
		(*e*) Describe any sensitivity analyses		The analyses performed are described above.
**Results**				
Participants	13*	(a) Report numbers of individuals at each stage of study—eg numbers potentially eligible, examined for eligibility, confirmed eligible, included in the study, completing follow-up, and analysed	8	47 participants were included in the cohort study. There were no loss to follow-up.
		(b) Give reasons for non-participation at each stage		Nothing to report.
		(c) Consider use of a flow diagram		
Descriptive data	14*	(a) Give characteristics of study participants (eg demographic, clinical, social) and information on exposures and potential confounders	8	Detailed description is found at page 8.
		(b) Indicate number of participants with missing data for each variable of interest		None
		(c) *Cohort study*—Summarise follow-up time (eg, average and total amount)	8	The athletes were monitored for a median of 42 days (IQR, 35-56). During the study period, the athletes performed a total of 1,500 (mean per athlete 32±19) exercise sessions and a total of 1,722 hours of exercise, of which 1,130 (65%) hours at low- to moderate intensity, 354 hours (21%) at high intensity and 238 hours (14%) at very high intensity.

				During the study period, the participants were continuously monitored with ICM for a total of 55,944 hours (2,331 days). The median duration of monitoring per athlete was 1,008 hours (IQR, 840-1,344)
Outcome data	15*	*Cohort study*—Report numbers of outcome events or summary measures over time	10	During the study period, we did not observe any episodes of VT or serious adverse clinical events. We did not observe any VT episodes or adverse clinical events during the 47 CPETs to maximum effort that were performed in the study. Supplementary Table S1 shows arrhythmia episodes recorded during 24-h ECG monitoring following CPET (in total 1,128 hours of monitoring). One athlete had two episodes at rest of non-sustained VT of four and six complexes, respectively, which weren't detected by the ICM due to no high-rate and the short duration. Four athletes had a total of four AF episodes. One athlete presented with >500 premature ventricular contractions (PVC) per 24 hours. We observed a total of 1,445 AF episodes (mean 31±3, median 3, IQR 0-21) among 31 (66%) of the

athletes in the study. Among athletes who experienced at least one AF episode during the study period, the mean number of episodes was 47±86 (median 17, IQR 3-33). There were 152 AF episodes (11%) with a duration >60 minutes and the median duration of AF episodes was 3 minutes (IQR 1.2-13.0, mean 32, SD 104). Fifteen episodes were classified as high-rate AF. Only one high-rate episode occurred during exercise, which was a low- to moderate intensity session.

In total, 13 individuals (28%) experienced recurrent AF during exercise, of whom 11 also had AF episodes unrelated to exercise. The median duration of AF episodes during exercise was 2.4 minutes (IQR 1.2-8.2, mean 20±55). Two participants experienced AF only during exercise. Each of them had only one single AF episode during the total study period in which they performed a total of 39 and 21 hours of exercise, respectively. In both cases, AF started during high-intensity exercise, however, none of the episodes were high-rate episodes.

Main results	16	(*a*) Give unadjusted estimates and, if applicable, confounder-adjusted estimates and their precision (eg, 95% confidence interval). Make clear which confounders were adjusted for and why they were included	The analyses were not confounder-adjusted due to the observational design.
		(*b*) Report category boundaries when continuous variables were categorized	Not relevant
		(*c*) If relevant, consider translating estimates of relative risk into absolute risk for a meaningful time period	Not relevant

Continued on next page

Other analyses	17	Report other analyses done—eg analyses of subgroups and interactions, and sensitivity analyses		Nothing to report
**Discussion**				
Key results	18	Summarise key results with reference to study objectives	13	In this prospective cohort study using continuous monitoring with ICM, we did not observe any severe adverse clinical events or episodes of VT during more than 1,700 hours of exercise in endurance athletes diagnosed with paroxysmal AF. The athletes performed almost 600 hours of high or very high intensity exercise, and the rate of recurrent AF episodes was not higher during or subsequently following exercise, compared to the monitoring periods without relation to exercise. Exercise did not trigger AF episodes with fast ventricular response.
Limitations	19	Discuss limitations of the study, taking into account sources of potential bias or imprecision. Discuss both direction and magnitude of any potential bias	15	The main limitations are the relatively small study size and the relatively short period of monitoring. A more prolonged monitoring period may have revealed more arrhythmia episodes and provided the study with more power to detect different rates of arrhythmia episodes between the categories of exercise and to further decipher the temporal association of recurrent AF episodes with exercise. On the other hand, the targeted cardiac examination, with both cardiac imaging, extended

continuous monitoring, CPET to maximal effort and ambulant ECG monitoring subsequently following CPET, represents a sufficient and highly specific assessment protocol for arrhythmia evaluation in athletes[22], with a presumably very high sensitivity to detect clinically relevant arrhythmia episodes. The inability of the ICM to detect tachycardia shorter than 12 complexes is another limitation, and shorter non-sustained VTs may have remained undetected. On the other hand, asymptomatic shorter episodes of non-sustained VT are considered less clinically relevant than longer episodes and episodes seen in relation to symptoms or pathological clinical findings. Importantly, because individuals with cardiovascular comorbidities were excluded from the study, the results should not be projected to other subgroups of AF patients or to athletes with AF and concomitant cardiovascular conditions. All the athletes participating in the study underwent cardiovascular risk assessment before entering the study, limiting the generalizability of the results to unselected cohorts of athletes. Due to the eligibility criteria, both asymptomatic individuals and highly symptomatic athletes awaiting ablation therapy

				may be underrepresented in the study. Moreover, only 3 women were included. Cardiac arrhythmias are 5- to 10-fold less common in female compared to male athletes, but larger studies with a more balanced female representation are needed to properly address gender differences in the risk related to continuing sports participation with paroxysmal AF.
Interpretation	20	Give a cautious overall interpretation of results considering objectives, limitations, multiplicity of analyses, results from similar studies, and other relevant evidence	16	During a total of >1,700 hours of exercise and almost 600 hours with high- or very high-intensity exercise, we observed no serious adverse clinical events or VT among endurance athletes with paroxysmal AF. The rate of AF recurrence was lower during or within three hours following exercise, compared to the monitoring periods unrelated to exercise. The results of the current study suggest that, in the short term, exercise of high- and very high intensity is safe in endurance athletes diagnosed with paroxysmal AF and no cardiovascular comorbidities.
Generalisability	21	Discuss the generalisability (external validity) of the study results	16	As mentioned above, all included athletes in the study underwent cardiovascular risk assessment before entering the study, limiting

			the generalizability of the results to unselected cohorts of athletes. Due to the eligibility criteria, both asymptomatic individuals and highly symptomatic athletes awaiting ablation therapy may be underrepresented in the study.
**Other information**			
Funding	22	Give the source of funding and the role of the funders for the present study and, if applicable, for the original study on which the present article is based	This work was supported by The Norwegian Health Association and Vestre Viken Hospital Trust, Norway. The funders do not have any role in the study design and do not have any role in the collection, management, analysis, and interpretation of data; or in the writing of the report; or decision to submit the report for publication.

*Give information separately for cases and controls in case-control studies and, if applicable, for exposed and unexposed groups in cohort and cross-sectional studies.

Note: An Explanation and Elaboration article discusses each checklist item and gives methodological background and published examples of transparent reporting. The STROBE checklist is best used in conjunction with this article (freely available on the Web sites of PLoS Medicine at http://www.plosmedicine.org/, Annals of Internal Medicine at http://www.annals.org/, and Epidemiology at http://www.epidem.com/). Information on the STROBE Initiative is available at www.strobe-statement.org.
